# Recombinant Human Bone Morphogenetic Protein-2 in Debridement and Impacted Bone Graft for the Treatment of Femoral Head Osteonecrosis

**DOI:** 10.1371/journal.pone.0100424

**Published:** 2014-06-23

**Authors:** Wei Sun, Zirong Li, Fuqiang Gao, Zhencai Shi, Qidong Zhang, Wanshou Guo

**Affiliations:** Centre for Osteonecrosis and Joint-Preserving & Reconstruction, China-Japan Friendship Hospital, Beijing, China; University of Texas Southwestern Medical Center, United States of America

## Abstract

The purpose of this study was to compare the clinical outcomes of impacted bone graft with or without recombinant human bone morphogenetic protein-2 (rhBMP-2) for osteonecrosis of the femoral head (ONFH). We examined the effect of bone-grafting through a window at the femoral head-neck junction, known as the “light bulb” approach, for the treatment of ONFH with a combination of artificial bone (Novobone) mixed with or without rhBMP-2. A total of 42 patients (72 hips) were followed-up from 5 to 7.67 years (average of 6.1 years). The patients with and without BMP were the first group (IBG+rhBMP-2) and the second group (IBG), respectively. The clinical effectiveness was evaluated by Harris hip score (HHS). The radiographic follow-up was evaluated by pre-and postoperative X-ray and CT scan. Excellent, good, and fair functions were obtained in 36, 12, and 7 hips, respectively. The survival rate was 81.8% and 71.8% in the first and second group, respectively. However, the survival rate was 90.3% in ARCO stage IIb, c, and only 34.6% in ARCO stage IIIa(P<0.05). It was concluded that good and excellent mid-term follow-up could be achieved in selected patients with ONFH treated with impacted bone graft operation. The rhBMP-2 might improve the clinical efficacy and quality of bone repair.

## Introduction

Non-traumatic osteonecrosis of the femoral head (ONFH) frequently affects patients between 20 and 50 years of age, and may be progressed to femoral head collapse within 1 to 5 years. Without an effective intervention during this period, a majority of the victims may also progress to hip osteoarthritis, resulting in hip arthroplasty [1]. Due to active patients, a wide range of etiologies, and a poorly understood pathogenesis, patients in this age group most likely require multiple revision procedures in their lifetime. Therefore, it is greatly important to preserve the femoral head in patients with this diagnosis, and delay or avoid the application of hip arthroplasty [2].

Although surgeons use many procedures to preserve the femoral head in patients with hip osteonecrosis, there is no consensus regarding the best procedure. Treatment of femoral head necrosis is divided into conservative treatment and surgical treatment. Conservative treatment includes hyperbaric oxygen, shock wave, electrical stimulation, and drugs, such as alendronate, lipid-lowering drugs, and low-molecular weight heparin. Surgical treatment includes core decompression, various types of osteotomy, vascularized and non-vascularized bone graft, and total hip arthroplasty [2]. In 1994, Rosenwasser et al initially brought forth the concept of the “Light Bulb procedure”, in which the necrotic lesion was replaced by the bone grafting via the window on the femoral head-neck junction without any damage to the joint cartilage [3]. This procedure provided strong structural support for the femoral head, amended the morphology of the femoral head to a certain extent, and averted the further collapse of the femoral head. Furthermore, it had several advantages, including: being a simple surgical technique, low incidence of complication, and short duration of operation.

In the present study, we reported the middle-term efficacy of treating ONFH with Light Bulb procedure and impacted bone grafting that were performed in our center. The clinical efficacy was also compared for the procedures with and without rhBMP-2 in order to explore the optimal indication for this technique.

## Patients and Methods

This study was approved by the Ethics Committee of China-Japan Friendship Hospital (2013-KY-29). Written consents were provided by the patients to be stored in the hospital database and be used for clinical research.

### 2.1 Clinical data

A total of 46 patients with non-traumatic ONFH (79 hips), undergoing debridement and impacted bone grafting (IBG), were included in this retrospective study between January 2004 and May 2006. A majority of the patients had a history of SARS and were treated with high-dose corticosteroids, at one point. All the operations were performed or supervised by the primary author. The patients were divided into two groups. The first group of patients (N = 20, 36hips; 9 men and 11 women) was subject to the standard background therapy (debridement+IBG) plus rhBMP-2 application, and was referred to as Group IBG+rhBMP-2. Involvement of bilateral hips, left hip, and right hip was observed in 15, 1, and 5 patients in this group, respectively. The patients were 23 to 49 years old (average of 31.1 years) at the time of the operation with a mean pre-operation Harris hip score (HHS) of 67.2±11.9. The second group of patients (N = 26, 43 hips; 11 men and 15 women) was subject to the standard background therapy (debridement+IBG) alone, and was referred to as Group IBG. Involvement of bilateral hips, left hip, and right hip was observed in 17, 3, and 7 patients in this group, respectively. The patients were 22 to 54 years old (average of 30.7 years) at the time of the operation with a mean pre-operation Harris hip score (HHS) of 68.1±10.9. All the patients were preoperatively evaluated with MRI (GE Sigma profile 1.5T T1WI and T2WI; and T2WI fat-suppressed or STIR sequence for some of the patients), posteroanterior, and frog lateral radiographs for the conditions of the both hips. A majority of the patients also underwent CT scanning and 2D reconstruction at the coronal and sagittal views for both hips. There were no discrepancy between the two groups in their general status, such as clinical course and historical treating. The addition of rhBMP-2 in the therapeutic regimen was random rather than a double-blind design.

### 2.2 Staging and typing

All the patients were graded and classified according to the system of the Association Research Circulation Osseous (ARCO) [4] and China-Japan Friendship Hospital (CJFH) classification systems [5], [6] into Stage 0 to IV and Types C to L. No stage I and type M ONFH were identified in this series (Table 1).

**Table 1:** Preoperative stages and types identified for the involved hips (hips, n).

Groups	ARCO stage			CJFH type			
	IIb	IIc	IIIa	C	L1	L2	L3
IBG+BMP_2_	4	21	11	4	15	1	16
IBG	6	18	19	6	20	3	14

### 2.3 Efficacy assessment [7]

The clinical efficacy was assessed based on the Harris hip score (HHS). Excellent, good, fair, and poor results were defined as Harris hip scores over 90, 80–89, 70–79, and below 70, respectively. The radiological evaluation was performed and graded based on the posteroanterior and frog lateral radiographs as follows: overall roundness of the femoral head; mild collapse with incongruity between the acetabulum and the femoral head; and moderate to severe collapse. The CT evaluation criteria for patients with available data included: complete or partial repair and subchondral bone fracture.

### 2.4 Surgical procedure

**Figure 1:**
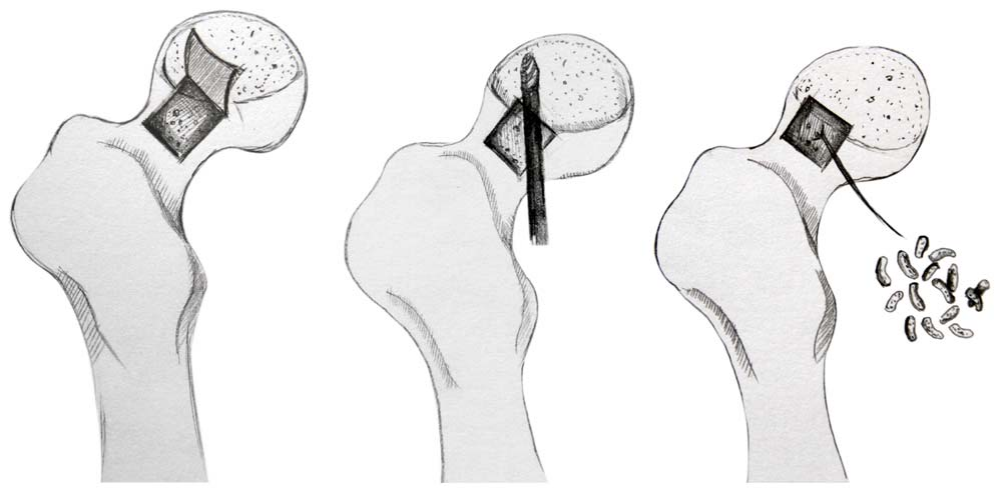
Schematic diagram of “Light Bulb procedure”: An approximate 1.5 cm×1.5 cm bone window was made at the femoral head–neck junction using osteotomes. And the necrotized bone located at anterolateral and upper side of the operated femoral head was alternately debrided using a high-speed drill. Then the cavity was filled with an autologous cancellous bone combination of rhBMP-2.

The procedure was performed with the patient lying in the lateral decubitus position. An incision of approximately 5–6 cm was made to the hip over the greater trochanter for an anterolateral approach (Watson-Jones approach), which was used to preserve the blood supply to the femoral head The fascia lata was split in the direction of the skin incision, and the anterior gluteus medius was detached. The anterior joint capsule was longitudinally spilt along the femoral head between the gluteus medius muscle and tensor muscle of fascia lata in order to expose the head-neck junction. A bone window (length and width of 1.5 cm) was made at the femoral head-neck junction using osteotomes. Under the monitoring of C-arm fluoroscopy, the necrotized bone located at anterolateral and upper side of the operated femoral head was alternately debrided using a high-speed drill and a curette, allowing the retention of at least 5 mm subchondral bone. For the sclerotic bone, multiple holes were created using a 3-mm drill until fresh blood was actively observed from the wound surface. Autologous cancellous bone, harvested from the iliac external circumferential lamella, was compressed layer by layer with the artificial bone (NOVOBone). The bone window was covered with the originally excised bone plate and was fixed with an absorbable screw. Four milligrams of rhBMP-2 (from Hangzhou Jiuyuang, China) was added for the patients of the first group. (Fig 1)

### 2.5 Post-operative treatment

After the surgery, all patients followed a strict rehabilitation and training program. Patients were maintained at toe-touch weight-bearing with two crutches for 12 weeks and were then advanced to approximately 50% weight-bearing for the second 12 weeks using a cane or crutch in the opposite hand. They began full weight-bearing as tolerated at six postoperative months. Higher impact loading activities (such as running) were begun at 12 postoperative months. The diphosphonate agent was prescribed for 6 to 12 months. After the operation, all patients were clinically and radiographically followed-up by the authors every three months during the first year and every six months for the following year. The clinical follow-up consisted of the determination of pre- and post-operative serial Harris hip scores, and serial AP, frog lateral radiographs and CT scan were used for the radiographic follow-up.

### 2.6 Statistical method

The data were analyzed using SPSS version 17.0 statistical software (SPSS Inc, Chicago, Illinois). Chi-square and Fisher's exact tests were used for statistical analysis of the data. P values of less than 0.05 were considered statistically significant.

## Results

### 3.1 Clinical results

With the exception of two patients (three hips) in IBG+BMP2 group and two patients (four hips) in the IBG group, all the patients received a follow up of 5 to 7.67 years (average of 6.1 years).

The clinical efficacy of the surgery between the two groups is summarized in Table 2. The efficacy of “excellent” and “good” was achieved in 36 and 12 hips, respectively, which accounted for 66.7% in all the operated hips (48/72). For the seven hips evaluated as “fair” in terms of clinical efficacy, the survival rate of the femoral head was 76.4% (55/72 patients). Seven of the 17 hips with “poor” efficacy (23.6%, 17/72 patients) had been previously subjected to total hip arthroplasty. The average post-operative score of all hips was 79.7±13.4 points at the last follow-up or just before conversion to a total hip implant.

**Table 2:** Clinical efficacy between the patients with and without BMP_2_.

Groups	Age	Harris scores		Clinical efficacy						
		Pre-operation	Post-operation	ARCO stage % (excellent/good)			CJFH type % (excellent/good)			
				IIb	IIc	IIIa	C	L1	L2	L3
IBG+BMP_2_ (n = 33)	31.1±8.7	67.2±11.9	82.3±13.2*	100%(4/4)	84.2%(16/19)*	30.0%(3/10)*	100% (4/4)	85.0% (17/20)	0.0% (0/1)	37.5%* (3/8)
IBG (n = 39)	30.7±8.4	68.1±10.9	78.9±12.6*	100%(6/6)	76.5%(13/17)*	37.5%(6/16)*	100% (6/6)	75.0% (9/12)	50.0%(1/2)	47.3%* (9/19)
*Total*	30.9±8.6	67.5±11.7	80.9±12.8*	100%(10/10)	80.6%(29/36)*	34.6%(9/26)*	100% (10/10)	81.3% (26/32)	33.3%(1/3)	44.4%* (12/27)

Note: * P<0.05 The statistical value was derived from chi-square test (no statistical value can be obtained from the Fisher's exact test); P<0.05 between IIb and IIc as well as IIc and IIIa; statistical difference was also found between Type L1 and Type L3, and there were significant statistical differences between pre-and post-operation Harris score, but no statistical differences between IBG+BMP2 and IBG groups.

As for the patients with IBG+BMP2, the following efficacy results were obtained: “excellent” and “good” in 17 and 6 hips, respectively (collaboratively accounting for 69.7% of the patients); “favorable” in 4 hips (81.8% of survival rate for femoral head); and “poor” in 6 hips (18.2%). The average post-operative score was 82.3±13.2 points.

As for the patients with IBG alone, the following efficacy results were obtained: “excellent” in 19 hips and “good” in 6 hips (collaboratively accounting for 64.1% of the patients); “favorable” in 3 hips (the survival rate of the femoral head: 71.8%); and “poor” in 11 hips (28.2%). The average postoperative score was 78.9±12.6 points (Table2).

No complications were observed during the operation; however, the postoperative complications included ectopic ossification in three hips (two in IBG+BMP2 and one in IBG group), and lateral femoral cutaneous nerve lesion in four hips (both 2 hips in the 2 groups).

### 3.2 Radiological results

**Figure 2:**
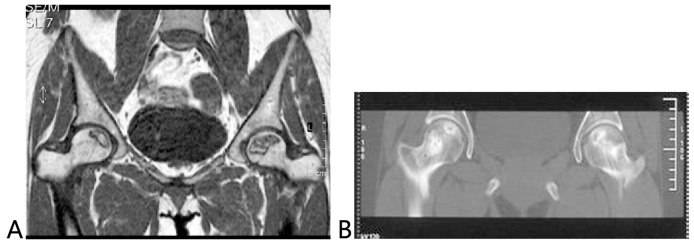
A 31-year-old patient with ONFH after SARS. A: Preoperative magnetic resonance image showed ARCO stage IIb/CJFH Type C (the right hip) and ARCO stage IIc/CJFH Type L1 ONFH (the left hip); B: This Computed tomography scan was performed at five years after the impacted bone grafting alone (without addition of BMP2) and showed the femoral head maintained its spherical shape an incomplete repair for the necrosis with the residual lesion at the subchondral bone of the femoral head.

**Figure 3:**
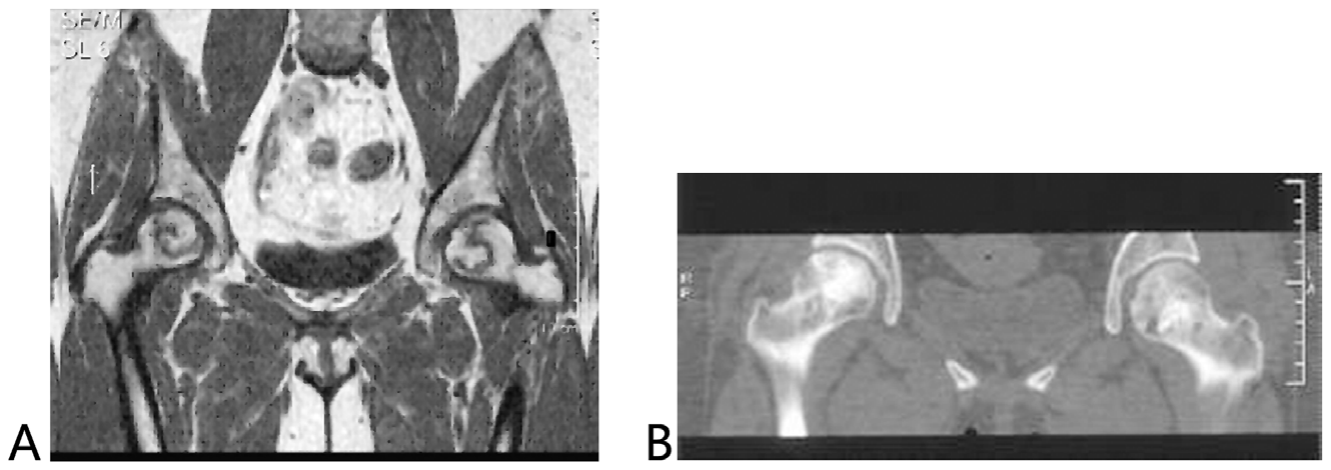
A 30-year-old patient with ONFH after SARS. A: Preoperative magnetic resonance image showed ARCO stage IIc/CJFH Type L3 (the right hip) and ARCO stage IIb/CJFH Type C ONFH (the left hip); B: This Computed tomography scan was performed five years after being treated with impacted bone grafting with the addition of rhBMP2 and showed the complete repair of the osteonecrotic region, the amount of sclerotic bone was more improved and the articular surface remains intact without collapse.

The radiological results were similar to those obtained from clinical evaluation. The roundness and complete repair of the femoral head was observed in 34 of the 36 hips with “excellent” outcomes. For a patient with ONFH (IIIa), who developed this condition after a high dose administration of corticosteroid against SARS, the femoral head was not round as shown in the radiogram, but the HHS score was at the “excellent” level (90) after the follow of 7.6 years. Of the 12 hips with “good” outcomes, non-roundness but no incongruity of the joint was found in seven hips, and roundness of the femoral head was found in five hips. In all of the 17 hips with “poor” outcomes, more than 4 mm femoral head collapse, incongruity of the joint, subchondral bone fracture (by CT), bone marrow edema (by MRI), and asymmetrical changes in the articular space were found, but without femoral head fracture. As detected by radiographic findings for the repair of the disease, the complete repair of the disease and the amount of sclerotic bone were more improved in patients with the addition of BMP2 as compared to those without such addition (Fig. 2,3).

## Discussion

Non-traumatic osteonecrosis of the femoral head is one of the major causes of disability in young patients. Symptomatic ONFH typically progresses with the collapse and incongruity of the joint. An effective treatment for ONFH is supposed to alleviate pain and maintain the shape of the femoral head in order to prevent any deterioration of the function of the hip joint. Various head-preserving procedures have been used for this disease in order to avert the need for a total hip arthroplasty. These methods include core decompression (CD), various types of osteotomies, and different methods of nonvascularised and vascularised bone grafting [2], [8]–[11].

None of these methods have been found to be universally successfull and there have been major variations in the clinical efficacy among different reports. CD is one of the methods most frequently utilized for the treatment of early stage ONFH, which could relieve the intraosseous pressure improving venous return and promoting the vascularization of the necrotic area of the femoral head. However, one of the possible causes for its failure is its separate use, which cannot supply enough of a ground for osteoanagenesis in the necrotic area [12]–[15]. The clinical and radiographical results of various osteotomies were indicated for cases with an intact load-bearing area of more than 40%. Currently, osteotomy is not widely accepted as the standard treatment of ONFH, because of its uncertain prognosis that can increase the difficulty of total hip arthroplasty [16]–[18]. Therefore, various bone grafting procedures including vascularised or non-vascularised bone grafting have been developed to directly influence the pathological process and to avert the application of hip arthroplasty [18]. Vascularised bone grafting, which has inconsistent long-term success rates, is also characterised by a prolonged operation, complicated techniques, major trauma, and a significant number of complications [19], [20]. Therefore, many researchers began to introduce non-vascularised bone grafting [21]. The literature has not demonstrated any particular superior procedure with respect to the operative treatment of hip osteonecrosis in terms of conversion to total hip arthroplasty or radiographic progression of disease.

For selected patients with ONFH, the local debridement and impacted bone grafting could achieve an excellent or good efficacy. This procedure was first introduced by Rosenwasser [3] and achieved a femoral head survival rate of up to 81% in 15 patients with non-traumatic ONFH who received a mean follow-up period of 12 years. This procedure was further modified by Mont et al. [21] who added demineralized bone matrix (DBM) into the bone graft and achieved an excellent-plus-good rate of 86% in 19 patients (21 hips) after a mean follow-up period of 4.5 years. The efficacy result obtained in this series (survival rate of the femoral head: 81.8%; excellent-plus-good rate: 66.7%), after a follow up period of more than 5 years, demonstrated the effectiveness of this procedure in spite of the slight inferiority of the results to those reported by the abovementioned authors. However, since the clinical efficacy of these procedures was not mentioned by the stage and type of the ONFH, these two reports had limited applicability in finding the optimized candidates for this procedure. The results obtained from this series suggested that this procedure could achieve a preferable efficacy in patients with ARCO IIONFH over those with ARCO III ONFH, and in patients with CJFH Types C and L1 ONFH over those with Types L2 and L3 ONFH. Therefore, we recommended an accurate stage and type data for a patient with ONFH to be a predominant factor for the success of a femoral head-preserving surgery. This procedure could be optimally indicated for the patients in stage ARCOII and those with Types C and L1 ONFH. However, one needs to be cautious for patients in stage IIIa and those with Type L3 ONFH unless the patient is young enough (≤35 years).

Both Animal and clinical studies have demonstrated that BMP-2, a member of the BMP family, improved the ossification activity by inducing the directional differentiation of mesenchymal stem cells to osteoblasts, thus promoting osseous healing and repair [22]–[25]. Lieberman [26] was the first researcher that mixed BMP, purified from human bones, with antigen-extracted lyophilized bone allograft and implanted the mixture using an impacted bone grafting via the window created at the junction of the femoral head and neck into 16 patients (17 hips) with ONFH who had been subject to debridement. In this effort, the femoral head was successfully preserved in 14 hips (86%) after a mean follow-up period of 53 months. For the 14 hips with successful surgery, a Ficat stage of II A (comparable to ARCO stage II) was identified for their condition. However, the remaining three hips, for which a Ficat stage of II B (comparable to ARCO stage III) was identified, were subject to a total hip arthroplasty due to failure of the primary surgery failure, suggesting that the success of the femoral head-preserving surgery could be closely related to the stage of the disease. As shown in the present study, a higher success rate and an excellent-plus-good rate were observed for the hip-preserving surgery in the patient with added rhBMP-2 as compared to those without (81.8% vs. 71.8% and 69.7% vs. 64.1%, respectively). Although no statistical difference was found, the proportion of the patients with a successful surgery was obviously improved. However, as shown by a further comprehensive analysis, the efficacy was improved only in patients with ARCO stage II or CJFH Types C and L1 ONFH but not in those with ARCO stage III or CJFH Types L3 disease. In combination with the radiological data, it was concluded that rhBMP2 was effective in improving the speed and quality of the bone repair inside the lesions, but was limited in the repair of the subchondral osteonecrosis and fracture (ARCO stage III), where the osteonecrosis occupied most of the subchondral bone (CJFH Type L3). Although the data regarding stages and types of ONFH were analyzed in this study, no statistical difference was obtained, probably due to the small patient size and the inclusion of a few patients with some specific types of ONFH. This was one of the two limitations of this study. The other limitation was that two kinds of artificial materials (Novobone and BMP2) were used during the study and were undistinguished from each other during the statistical analysis, which could have had an influence on the results due to their different ossification mechanisms.

The method for rhBMP-2 application still needs to be modified. In previous reports, rhBMP-2 was evenly implanted after it was incorporated in the artificial or allograft bone. Several animal studies showed that the repair mainly occurred at the edge of the femoral head defects, where the blood supply was observed rather than the center of the defects, in which the unrepaired fibrous tissue was located [27], [28]. Therefore, an artificial or allograft bone mixed with highly concentrated rhBMP-2, implanted into the subchondral bone plate and the rim of the defect, might allow for a more rapid ossification at the implanted site in order to strengthen the subchondral bone plate and to enhance the compressive strength of the necrotized femoral head, while the addition of rhBMP-2 could be unnecessary to other sites.

As reported by Vandermeer et al. [29], concurrent implantation of diphosphate and rhBMP-2 achieved an optimal outcome for the repair of the necrotized femoral head in animals. This observation indicated that a concurrent implementation of suppression to osteolysis and promotion to ossification might facilitate a better necrotized bone repair. This further supported the application of this therapy in the treatment of ONFH, suggesting that a combined implantation of diphosphate and rhBMP-2 might allow for a more satisfactory repair of the necrotized femoral head [30]. Recent studies showed that the off-label use of a high dose (more than 40 mg) of rhBMP-2 was associated with an increased risk of new cancer. We should therefore, further evaluate the potential risk of cancers in patients receiving high-dose rhBMP2 [31], [32].

## Conclusions

In summary, mid-term excellent and good functions could be achieved in the selected patients (ARCO stage II or CJFH Types C and L1) with impacted bone graft operation through a window at the femoral head-neck junction. This procedure was characterised by the following superiorities of simple surgical technique, a minimally invasive surgery and low incidence of complication, and might be effective at avoiding or forestalling the need for hip replacement in younger patients with early stage of femoral head osteonecrosis Although no statistical difference was obtained in clinical results, from the radiological data, it could be concluded that rhBMP2 might improve the speed and quality of the bone repair inside the lesions. The lack of statistical significance could have been due to the small patient size and the presence of a few patients with some specific types of ONFH.
